# Source–Sink Manipulation Affects Accumulation of Zinc and Other Nutrient Elements in Wheat Grains

**DOI:** 10.3390/plants10051032

**Published:** 2021-05-20

**Authors:** Lan Wang, Haiyong Xia, Xiaojing Li, Yuetong Qiao, Yanhui Xue, Xilong Jiang, Wei Yan, Yumin Liu, Yanfang Xue, Lingan Kong

**Affiliations:** 1 Crop Research Institute, Shandong Provincial Key Laboratory of Crop Genetic Improvement, Ecology and Physiology, Shandong Academy of Agricultural Sciences, Jinan 250100, China; wl96abc@163.com (L.W.); lixiaojing4306@163.com (X.L.); qiaoyt126@163.com (Y.Q.); yanhuixuesaas@163.com (Y.X.); jiangxilong0513@126.com (X.J.); kongling-an@163.com (L.K.); 2 College of Life Sciences, Shandong Normal University, Jinan 250014, China; 3 College of Agronomy, Qingdao Agricultural University, Qingdao 266109, China; 4 College of Resources and Environmental Sciences, China Agricultural University, Beijing 100193, China; 5 Maize Research Institute, National Engineering Laboratory of Wheat and Maize, Shandong Academy of Agricultural Sciences, Jinan 250100, China; weiysdl@163.com (W.Y.); xyfang198692@163.com (Y.X.); 6 Institute of Agricultural Resources and Environment, Shandong Academy of Agricultural Sciences, Jinan 250100, China; liuyumin666@126.com

**Keywords:** photoassimilate, micronutrient, macronutrient, phytohormone, biofortification, phytate, bioavailability

## Abstract

To better understand the source–sink flow and its relationships with zinc (Zn) and other nutrients in wheat (*Triticum aestivum* L.) plants for biofortification and improving grain nutritional quality, the effects of reducing the photoassimilate source (through the flag leaf removal and spike shading) or sink (through the removal of all spikelets from one side of the spike, i.e., 50% spikelets removal) in the field of the accumulation of Zn and other nutrients in grains of two wheat cultivars (Jimai 22 and Jimai 44) were investigated at two soil Zn application levels. The kernel number per spike (KNPS), single panicle weight (SPW), thousand kernel weight (TKW), total grain weight (TGW) sampled, concentrations and yields of various nutrient elements including Zn, iron (Fe), manganese (Mn), copper (Cu), nitrogen (N), phosphorus (P), potassium (K), calcium (Ca) and magnesium (Mg), phytate phosphorus (phytate-P), phytic acid (PA) and phytohormones (ABA: abscisic acid, and the ethylene precursor ACC: 1-aminocylopropane-1-carboxylic acid), and carbon/N ratios were determined. Soil Zn application significantly increased the concentrations of grain Zn, N and K. Cultivars showing higher grain yields had lower grain protein and micronutrient nutritional quality. SPW, KNPS, TKW (with the exception of TKW in the removal of half of the spikelets), TGW, and nutrient yields in wheat grains were most severely reduced by half spikelet removal, secondly by spike shading, and slightly by flag leaf removal. Grain concentrations of Zn, N and Mg consistently showed negative correlations with SPW, KNPS and TGW, but positive correlations with TKW. There were general positive correlations among grain concentrations of Zn, Fe, Mn, Cu, N and Mg, and the bioavailability of Zn and Fe (estimated by molar ratios of PA/Zn, PA/Fe, PA × Ca/Zn, or PA × Ca/Fe). Although Zn and Fe concentrations were increased and Ca was decreased in treatments of half spikelet removal and spike shading, the treatments simultaneously increased PA and limited the increase in bioavailability of Zn and Fe. In general, different nutrient elements interact with each other and are affected to different degrees by source–sink manipulation. Elevated endogenous ABA levels and ABA/ACC ratios were associated with increased TKW and grain-filling of Zn, Mn, Ca and Mg, and inhibited K in wheat grains. However, the effects of ACC were diametrically opposite. These results provide a basis for wheat grain biofortification to alleviate human malnutrition.

## 1. Introduction

Zinc (Zn) is an essential micronutrient to sustain the nutritional health of plants, animals and humans [1,2]. According to reports, more than one third of the world’s population is suffering from potential Zn deficiency [3,4,5]. Wheat (*Triticum aestivum* L.) is an important global cereal crop, accounting for about 30% of human daily calorie intake [6]. The grain Zn concentration in wheat plants is generally low, averaging around 28–32 mg·kg^−1^ globally [7,8], which is far lower than the biofortification target value of 3–50 mg·kg^−1^ recommended by the Food and Agriculture Organization of the United Nations (FAO, Rome, Italy), the HarvestPlus project from the Bill & Melinda Gates Foundation and the World Health Organization (WHO, Geneva, Switzerland) [9,10]. It is therefore of great interest to biofortify wheat grains with Zn to alleviate global malnutrition.

The source–sink relationship is the basis for grain yield and nutrient concentration formation [11]. Source–sink regulation involves the absorption/production, distribution, transport, transformation and accumulation of photoassimilates and nutrient elements, as well as the interaction between organs, and the coordination of this process is the prerequisite for high yield of crops [12]. Source–sink interactions have been intensively investigated for nearly a century to improve crop yield potential, but less research has been conducted on grain nutritional quality [13,14]. Reducing the sources of carbohydrates from photosynthesis through spike shading or defoliation or reducing the sink size of grain through partial spikelet removal has been commonly conducted to investigate the source–sink limitations of crop assimilates for grain development and dry matter accumulation [15,16]. These physical manipulations were recently applied to investigate the source–sink relationship of micronutrient accumulation in wheat grains, especially for Zn [17,18,19]. In these experiments, the results indicate that Zn and dry matter accumulation in wheat grains is restricted by sink capacity and source supply, but the effects of reducing sink capacity or source supply on grain Zn concentrations are inconsistent. For example, both Zhang et al. [17] and Xia et al. [19] observed that defoliation by removing all of the leaf blades from tagged culms reduced the source-to-sink ratio and decreased grain Zn concentrations accordingly, but in another study undertaken by Zhang et al. [18], defoliation increased the grain Zn concentration. Partial spikelet removal and spike shading increased grain Zn concentrations in the studies of Zhang et al. [17,18], but led to decreases in the treatment of spike shading in Xia et al. [19]. Grain size and number are important factors determining the source–sink relationship of wheat plants. As reported by previous studies [20,21,22,23,24,25,26], there are inconsistent conclusions about whether the Zn concentrations depend on the grain size and number. No correlation between the grain Zn concentration and thousand grain weight was found by Velu et al. [23] in adapted wheat lines, indicating no concentration effect resulting from the grain size. A significant negative correlation in unadapted wheat was reported by Morgounov et al. [20], but a significant positive correlation was observed in our previous study [26]. Grain Zn concentrations were correlated with grain numbers per m^2^ [20] or per pot [25], or spike numbers [26] negatively, but not with kernel numbers per spike [26]. Such contradictory and inconsistent results possibly result from the different genotypes of wheat cultivars investigated or different environmental conditions, which need to be further verified across multiple location-years and more wheat varieties to better understand the source–sink relationships of grain Zn accumulation, especially regarding the impacts of artificial source–sink manipulation on grain Zn accumulation and its interaction with the photosynthate transport.

The flag leaf of wheat, as the main source of photoassimilate, has been reported to contribute to more than 50% of grain filling, showing the higher importance of the flag leaf than other leaves to the source–sink relationship, whereas its defoliation decreased grain yield by 18–30%, suggesting the role of other lower leaves to yield increased when the flag leaf was shaded or removed [27]. Similarly, the source of micronutrients such as Zn and iron (Fe) in the wheat grain depends mainly on the flag leaf and, to a lesser extent, on the lower leaves [28]. Most previous studies on the effect of defoliation on grain Zn accumulation of wheat were conducted by removing all of the leaf blades from tagged culms [17,18,19]. However, the effect of only the flag leaf removal on the source–sink relationship of Zn accumulation in wheat grains and the role of other lower leaves are unclear and have been less extensively investigated.

Various phytohormones coordinate the source–sink relationship within the crop plant, with abscisic acid (ABA)-based chemical signaling showing determinant roles in promoting the leaf senescence and grain-filling [13,29]. Senescence-associated mRNAs have been induced by exogenous ABA application, and thus accelerated leaf senescence [30]. ABA levels appear to be increased during senescence affected by drought or heat [31]. Yang and Zhang [13] found that elevated wheat endogenous ABA levels and higher ratios of ABA/gibberellins (GAs) and ABA/ethylene were necessary for efficient grain-filling. Applying exogenous ABA at a low concentration or moderate soil-drying after anthesis increased the endogenous ABA level, which can improve the activities of key enzymes involving carbohydrate metabolism in stem and/or grain, increase the loading and unloading capacity of assimilates, and finally promote remobilization of assimilates to wheat grains and accelerate grain starch synthesis [13]. Although hormone signaling, root and leaf growth and senescence, Zn uptake, transport and remobilization, and kernel development are intrinsically linked during grain-filling of wheat in theory [14,32], the relationship between ABA and grain Zn accumulation in wheat plants has not been observed or established.

The bioavailability of Zn in wheat grains is also an important factor affecting human intake of Zn. Wheat grain is rich in anti-nutritional compounds such as phenolic compounds and especially phytic acid (PA) that reduce the biological availability of Zn in the digestive tract [33], and the human body lacks phytase. Therefore, the molar ratio of PA/Zn is often applied to evaluate the bioavailability of Zn in wheat-based food [34,35]. Ca^2+^ can enhance the binding ability of PA and Zn^2+^, forming a phytic acid-calcium (Ca)-zinc complex, so the molar ratio of PA × Ca/Zn can better predict the bioavailability of Zn [36]. According to the WHO [37], the critical value of the molar ratio of PA/Zn that affects Zn absorption is 15. When the value exceeds 15, the bioavailability is only 10–15%, and Zn absorption will be severely inhibited; when it is below 15, it can represent 30–35% Zn availability; and only when less than 5 does it have no effect on Zn absorption. A molar ratio of PA × Ca/Zn below the critical value of 200 suggests good Zn bioavailability [36]. Similarly, the molar ratio of PA/Fe and/or PA × Ca/Fe has also been calculated to estimate Fe bioavailability in wheat grains, and the critical value of PA/Fe molar ratio is 10 [38]. Therefore, the content of PA and the molar ratio of PA/Zn or PA/Fe in wheat grains are the key factors affecting the absorption and utilization of Zn. Reducing the PA content and the molar ratio of PA/Zn or PA/Fe is a feasible means to improve the bioavailability of Zn/Fe in wheat food. However, the effects of physical source–sink manipulations through defoliation, spike shading or spikelet removal on bioavailability of Zn and Fe in wheat grains have never been investigated.

In addition to Zn, micronutrient elements, such as Fe, manganese (Mn) and copper (Cu), also play important roles in crop yield and quality and human nutrition. More than 60% of the world’s population is Fe-deficient, and the deficiency of Cu is also common in developing countries [39]. It is well-known that, in addition to micronutrients, crop plants have a large demand for carbon (C), nitrogen (N), phosphorus (P), potassium (K), Ca and magnesium (Mg) to sustain normal growth. There have been some reports that the supply of exogenous carbohydrate, N, P, K and other nutrients (including the Irving–Williams series metals), and their status within the plant affects Zn accumulation in wheat grains [14,40,41]. At present, most studies on source–sink manipulations focus on their effects on the micronutrient accumulation in wheat grains, but less on the grain macro-elements (C, N, P, K, Ca and Mg). Consequently, there is a lack of systematic understanding of the changes to macro- and micronutrients and the cross-talks between Zn and C, N, P, K or other divalent cations in wheat grains.

In this study, we reduced the source of photosynthesis through flag leaf removal and spike shading or reduced the total grain sink by 50% spikelet removal in two wheat cultivars under different soil Zn application levels, and investigated their effects on (1) grain yields and yield components including the total grain weight (TGW) sampled, single panicle weight (SPW), kernel number per spike (KNPS) and the thousand kernel weight (TKW); (2) grain micronutrient accumulation including Zn, Fe, Mn and Cu; (3) changes in grain macro-elements (N, P, K, Ca, Mg, C/N ratio, phytate-P) and bioavailability of Zn and Fe (estimated by molar ratios of PA/Zn, PA/Fe, PA × Ca/Zn and PA × Ca/Fe) in wheat grains; (4) changes in phytohormones (i.e., ABA, the ethylene precursor ACC: 1-aminocylopropane-1-carboxylic acid and ABA/ACC ratio) in wheat grains at maturity; and (5) relationships among the above-mentioned grain yield and nutritional traits, and phytohormones across different wheat cultivars and soil Zn application rates. The differences in various grain traits between different wheat genotypes were also investigated. These results will provide a better systematic understanding of the source–sink relationship of Zn and other nutrient elements for wheat grain biofortification to alleviate malnutrition.

## 2. Results

### 2.1. Grain Yields and Yield Components

Soil Zn application had non-significant impacts on single panicle weights, kernel number per spike, thousand kernel weights and total grain weights of these two wheat cultivars (Table 1). The single panicle weight, kernel number per spike and total grain weight of Jimai 22 were significantly higher than those of Jimai 44, with increases of 13.3%, 14.5% and 12.2%, respectively. No significant effects of cultivars on the thousand kernel weight were observed. The single panicle weight, kernel number per spike and total grain weight were highest in the control treatment, followed by flag leaf removal secondly, spike shading thirdly, and half spikelet removal lastly. The single panicle weight, kernel number per spike and total grain weight most significantly decreased from 2.2 g in the control to 1.3 g in the treatment of half spikelet removal by 40.9%, from 38.6 to 19.4 g by 49.7%, and from 52.2 to 29.0 g by 44.4%, respectively. Among different source–sink treatments, the thousand kernel weights varied from 37.5 to 49.7 g and were in the order of “half spikelet removal” > “control” > “flag leaf removal” > “spike shading”. Spike shading had the lowest value, which was significantly lower than the control, and half spikelet removal had the maximum thousand kernel weight, which was significantly higher than the control (Table 1). In addition, as ANOVA indicated, the interaction of cultivars × source–sink manipulations significantly affected single panicle weights, and for the kernel number per spike, a significant interaction was found between treatments of soil Zn applications and source–sink manipulations (Table S1).

### 2.2. Grain Zn, Fe, Mn and Cu Concentrations and Yields

The grain Zn concentration increased from 41.1 to 43.2 mg·kg^−1^ after applying Zn to the soil, with a significant increase of 5.1% (Table 2). However, the grain Cu concentration was significantly reduced from 5.6 to 5.3 mg·kg^−1^. Grain Zn, Fe, Mn and Cu concentrations of Jimai 22 were 7.1%, 5.7%, 5.1% and 12.1% lower than those of Jimai 44, respectively. In the source–sink treatments, the grain Zn, Fe, Mn and Cu concentrations varied dramatically from 35.8 to 53.2 mg·kg^−1^, from 42.1 to 52.8^−1^, from 39.0 to 59.6 mg·kg^−1^, and from 4.9 to 6.0 mg·kg^−1^, respectively, with the highest values observed in the treatment of half spikelet removal (except for Cu). Both half spikelet removal and spike shading treatments were associated with relatively higher micronutrient values than those of control and flag leaf removal, with the exception of Mn in spike shading. Compared with the control, half spikelet removal increased grain Zn, Fe and Mn concentrations by up to 48.6%, 25.4% and 31.3%, respectively, and spike shading increased grain Cu concentration by up to 22.4%. For Zn, Fe, Mn and Cu, the flag leaf removal only decreased grain Mn concentration significantly (Table 2). Grain Zn concentrations were significantly affected by the interactions of soil Zn applications × cultivars, cultivars × source–sink manipulations, and soil Zn applications × cultivars × source–sink manipulations (Table S2). Interactions of soil Zn applications × cultivars and cultivars × source–sink manipulations on grain Cu concentrations were also significant.

There were no significant impacts of soil Zn application or cultivars on grain Zn, Fe, Mn and Cu yields (Table S3). For source–sink treatments, grain Zn, Fe, Mn and Cu yields were all decreased compared with the control. The grain Mn yields in spike shading and Cu yields in half spikelet removal were most significantly reduced. The interaction of cultivars × source–sink manipulations significantly affected grain Zn and Mn yields (Table S3).

### 2.3. Concentrations and Yields of N, P, K, Ca, Mg and Phytate-P, C/N Ratios, and Molar Ratios of PA/Zn, PA/Fe, PA × Ca/Zn and PA × Ca/Fe in Wheat Grains

Compared to zero Zn supply, 30 kg ZnSO_4_·7H_2_O ha^−1^ significantly increased grain N and K concentrations from 17.1 to 19.2 g·kg^−1^, and from 3.8 to 4.3 g·kg^−1^, respectively (Table 3). Similar results were found in grain N and K yields (Table S3). Variation in Zn supply had non-significant impacts on other nutritional traits in wheat grains. Jimai 44, as a high-quality strong gluten wheat cultivar, had significantly higher grain N concentration, and significantly lower grain K and Ca concentrations, grain N, K, Ca, Mg and phytate-P yields, and molar ratios of PA/Zn, PA/Fe, PA × Ca/Zn and PA × Ca/Fe than those of the high-yielding Jimai 22 (Table 3 and Table S3).

Compared with the control, flag leaf removal significantly decreased the grain N concentration, but significantly increased the grain Mg concentration (Table 3). Both half spikelet removal and spike shading significantly increased grain N, P and phytate-P concentrations compared to the control and flag leaf removal treatment, with maximum values in half spikelet removal, but significantly reduced C/N ratios and molar ratios of PA × Ca/Zn, with minimum values in spike shading. In addition, grain Ca concentrations and molar ratios of PA/Zn, PA/Fe and PA × Ca/Fe in treatments of half spikelet removal and spike shading were all relatively lower than the control and flag leaf removal treatment. Compared with the control and flag leaf removal, grain K concentration was significantly decreased by half spikelet removal, but significantly increased by spike shading. Grain Mg concentration was significantly enhanced by half spikelet removal compared to the control and flag leaf removal treatment, but significantly decreased by spike shading compared to the treatment of flag leaf removal (Table 3). In contrast to the results of grain N, P, K, Ca, Mg and phytate-P concentrations, the corresponding yields were all decreased by source–sink treatments compared with the control (Table S3). Grain K and Ca yields in half spikelet removal, and Ca and Mg yields in spike shading, were most significantly reduced (Table S3).

The interaction of cultivars × source–sink manipulations significantly affected grain K concentrations and yields (Tables S2 and S3). The fertilizer Zn applications × source–sink manipulations interaction and the cultivars × source–sink manipulations interaction significantly affected grain Ca concentrations and yields (Tables S2 and S3).

### 2.4. Concentrations and Yields/Accumulation of ABA and ACC in Wheat Grains

Soil Zn application significantly increased the ABA concentration from 25.2 to 31.0 ng g^−1^, and the ratio of ABA/ACC from 0.7 to 1.0, but significantly decreased the ACC concentration and yield from 41.0 to 35.3 ng g^−1^ and from 1529.6 to 1322.2 ng, respectively (Table 4 and Table S4). The ACC concentration and yield of Jimai 44 was significantly higher than those of Jimai 22, but the results of ABA yield and ABA/ACC ratio were significantly lower than those of Jimai 22. Analysis of variance revealed significant effects of source–sink treatments on grain concentrations of ABA (15.4–42.6 ng g^−1^) and ACC (30.9–52.7 ng g^−1^), grain yields of ABA (510.8–1485.8 ng) and ACC (875.5–1741.7 ng), and the ratios of ABA/ACC (0.3–1.5). Compared with the control, the grain ABA concentration and ABA/ACC ratio were significantly increased by half spikelet removal, but decreased by spike shading. Spike shading significantly increased the grain ACC concentration. Grain ABA and ACC yields were all reduced by source–sink manipulations as compared with the control, with the ABA yield in spike shading and ACC yield in half spikelet removal decreased most significantly. The interaction of cultivars × source–sink treatments significantly affected grain ABA yields. Grain ACC concentrations and yields were significantly affected by the interaction of fertilizer Zn supply × cultivars (Table 4 and Table S4).

### 2.5. Relationships among Grain Yield Traits and Nutritional Quality-Related Parameters

**Figure 1:**
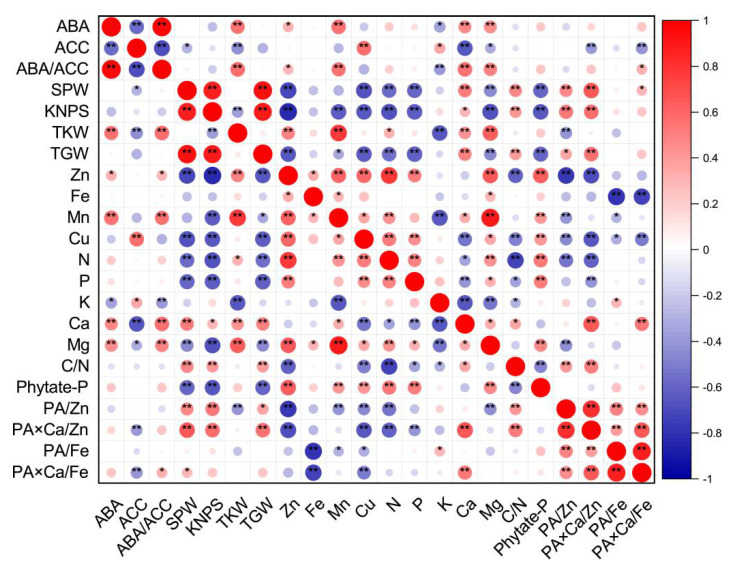
Corrplot representing correlation among measured grain phytohormones (concentrations of ABA and ACC, and ratios of ABA/ACC), grain yield traits and nutritional parameters (concentrations and ratios) of wheat crop across different soil Zn applications, cultivars and source–sink treatments (*n* = 48). Positive correlations are displayed in red and negative correlations are displayed in blue. The color legend on the right hand side of corrplot shows correlation coefficients and the corresponding colors. The intensity of the color and the circle size are proportional to the correlation coefficients. “*” and “**” indicate significant correlations at *p* ≤ 0.05 and *p* ≤ 0.01, respectively. The abbreviations are as follows: single panicle weight (SPW), kernel number per spike (KNPS), thousand kernel weight (TKW), total grain weight (TGW), concentrations of abscisic acid (ABA), the ethylene precursor 1-aminocylopropane-1-carboxylic acid (ACC), Zn, Fe, Mn, Cu, N, P, K, Ca, Mg and phytate-P, ratios of C/N and ABA/ACC, and molar ratios of phytic acid (PA)/Zn, PA × Ca/Zn, PA/Fe and PA × Ca/Fe in wheat grains.

Figure 1 shows a graphical display of the correlation matrix by corrplot. Considering all 48 data points in this study, the SPW was positively correlated with the KNPS and TGW (Figure 1). The KNPS was negatively correlated with TKW, but positively correlated with TGW. Single panicle weights, kernel numbers per spike or total grain weights were all negatively correlated with grain concentrations of Zn, Mn, Cu, N, P, Mg and phytate-P (with an exception of single panicle weight and Mn), but positively correlated with grain Ca concentration, C/N ratios, or molar ratios of PA/Zn, PA × Ca/Zn and PA × Ca/Fe (with the exception of KNPS/TGW and PA × Ca/Fe). TKW was positively correlated with grain Zn, Mn, N, Ca or Mg concentration, but negatively correlated with grain K concentration and the PA/Zn molar ratio (Figure 1).

Grain concentrations of Zn, Fe, Mn, Cu, N and Mg were all positively correlated with each other, except for Fe and Cu and Fe and N. Grain Zn and Cu concentrations were all positively correlated with grain P or phytate-P concentrations, but negatively correlated with C/N ratios. There were significant and positive correlations between grain Mn and Ca or phytate-P concentrations, and significant and negative correlations between grain Mn and K concentrations and between grain Cu and Ca concentrations. Most correlations between grain micronutrient concentrations, including Zn, Fe, Mn and Cu, and molar ratios of PA/Zn, PA × Ca/Zn, PA/Fe, or PA × Ca/Fe were negative (Figure 1).

For macronutrients N, P and K, only grain N concentrations were positively correlated with P, and grain N and P concentrations were all positively correlated with grain Mg and phytate-P concentrations, but negatively correlated with grain Ca concentrations, C/N ratios and molar ratios of PA × Ca/Zn. Grain N concentrations were negatively correlated with molar ratios of PA/Zn. Grain K concentrations were negatively correlated with grain Ca and Mg concentrations, and C/N ratios, but positively correlated with molar ratios of PA/Fe (Figure 1).

There were significant and positive correlations between grain Ca concentrations and grain Mg concentrations, C/N ratios, or molar ratios of PA × Ca/Zn and PA × Ca/Fe, between grain Mg and phytate-P concentrations, and between C/N ratios and molar ratios of PA/Zn or PA × Ca/Zn. There were significant and negative correlations between grain Mg concentrations and molar ratios of PA/Zn, and between C/N ratios and grain phytate-P concentrations. In addition, positive correlations were observed among molar ratios of PA/Zn, PA × Ca/Zn, PA/Fe, and PA × Ca/Fe (Figure 1).

As shown in Figure S1, there were negative correlations between the TKW and grain K yield, between grain Zn yields and molar ratios of PA/Zn, and between grain Fe yields and molar ratios of PA/Fe or PA × Ca/Fe. Non-significant correlations were observed between the TKW and grain yield of Fe, Cu, N, P, Ca, or phytate-P, between grain Fe and P yield, between grain K and Zn, Fe, or Mn yield, between the C/N ratio and grain yield of Zn, Fe, Cu, N, P, K, phytate-P or TKW, between the molar ratio of PA/Zn and grain yield of Fe, Mn, Cu, N, P or Mg, and between the molar ratio of PA × Ca/Zn and grain yield of Zn, Fe, Cu, P or K. Except for negative correlations between the grain Fe yield and the molar ratio of PA/Fe or PA × Ca/Fe and positive correlations between the molar ratio of PA × Ca/Fe and grain yield of Ca or phytate-P, there were non-significant correlations between the molar ratios of PA/Fe or PA × Ca/Fe and other grain nutritional traits. The correlations for other values presented were all positive (Figure S1).

### 2.6. Relationships between Grain Phytohormones and Grain Yield Traits or Nutritional Quality-Related Parameters

The Pearson correlation showed that grain ABA concentrations and ratios of ABA/ACC were positively correlated with parameters of TKW, grain concentrations of Zn, Mn, Ca, Mg, and molar ratios of PA × Ca/Fe (except for ABA and PA × Ca/Fe), but negatively correlated with grain K concentrations (Figure 1). However, grain ACC concentrations were negatively correlated with SPW, TKW, grain Ca and Mg concentrations, and molar ratios of PA × Ca/Zn or PA × Ca/Fe, and positively correlated with grain Cu and K concentrations (Figure 1). Grain ABA yields and ABA/ACC ratios were all positively correlated with TKW, grain Mn yields and PA × Ca/Fe molar ratios (Figure S1). In addition, grain ABA yields were positively correlated with SPW, TGW, grain Zn, N, P, Ca, Mg and phytate-P yields, and molar ratios of PA × Ca/Zn. Grain ACC yields were positively correlated with SPW, KNPS, TGW, and grain Cu, N and K yields, but negatively correlated with TKW (Figure S1). In terms of concentrations or yields, there were positive correlations between ABA and ABA/ACC, and negative correlations between ABA and ACC and between ACC and ABA/ACC (Figure 1 and Figure S1).

### 2.7. Principle Component Analysis (PCA) of Various Parameters of Wheat Affected by Source–Sink Manipulation

**Figure 2:**
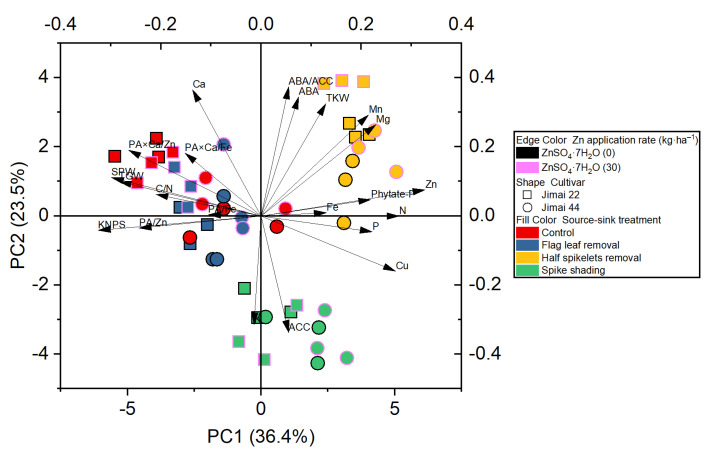
Principle component analysis (PCA) of the effect of source–sink manipulation on various investigated parameters of wheat plants. The abbreviations are as follows: single panicle weight (SPW), kernel number per spike (KNPS), thousand kernel weight (TKW), total grain weight (TGW), concentrations of abscisic acid (ABA), the ethylene precursor 1-aminocylopropane-1-carboxylic acid (ACC), Zn, Fe, Mn, Cu, N, P, K, Ca, Mg and phytate-P, ratios of C/N and ABA/ACC, and molar ratios of phytic acid (PA)/Zn, PA × Ca/Zn, PA/Fe and PA × Ca/Fe in wheat grains.

The principle component analysis revealed the distribution of the different treatments performed on the wheat crop, and showed a better visualization of the relationships and great variation present among all the investigated parameters (Figure 2). The results obtained from the plot of PCA performed for two factors (cumulative variance, 59.9%) show that the first factor explains 36.4% of the variation, while 23.5% of the differences are explained by the second factor (Figure 2).

## 3. Discussion

### 3.1. Effects of Soil Zn Fertilization on Grain Yield Traits and Nutrient Accumulation of Wheat

In our study, soil Zn application did not affect the yield traits of wheat (SPW, KNPS, TKW or TGW) or the accumulation of most nutrient elements significantly, indicating that Zn was not a growth limiting factor at the experimental site (Table 1, Table 2 and Table 3 and Tables S1–S3). Similarly, in the study of Zhang et al. [42], the thousand kernel weight, harvest index and grain yield of winter wheat were unaffected by soil or foliar Zn applications. Zhao et al. [43,44] also found that regardless of the application method (soil or foliar alone, soil + foliar) and form (Zn-EDTA or ZnSO_4_), Zn fertilization had a non-significant effect on the grain yield. These results might be firstly due to the relatively high soil DTPA-Zn and thus good Zn nutritional status in wheat [45]. Here, the soil DTPA-Zn was 1.6 mg·kg^−1^, much higher than the reported critical value of 0.46–0.75 mg·kg^−1^, below which wheat responds to Zn application [45,46], and no visible Zn deficiency symptoms during the whole wheat growth period were observed. Therefore, Zn fertilization via soil had no significant effect on the grain yield traits of wheat in the present study. Similar results were also observed on a larger scale by Zou et al. [47], who investigated the biofortification of wheat with Zn through soil, foliar or combined (soil + foliar) Zn fertilization over 23 experimental site-years in seven countries (China, India, Kazakhstan, Mexico, Pakistan, Turkey and Zambia). The significant grain yield increase due to soil Zn fertilization was found during all six experimental site-years only in Pakistan, and not in any other countries. Across all cropping years and locations, soil Zn fertilization led to a yield increase of only 5.1% [47]. Secondly, the lack of significant differences, particularly in the grain yield traits, may be attributed to the uneven spread of Zn fertilizer and insufficient sample numbers in our research, in which only 28 wheat spikes of each plot were collected for analysis, particularly under field conditions. In contrast to the above-mentioned results obtained under real field conditions, a well-controlled pot experiment showed that Zn fertilizer application to soil significantly increased weight per grain and total wheat grain yield and in both experimental years and both wheat cultivars [48].

**Figure 3:**
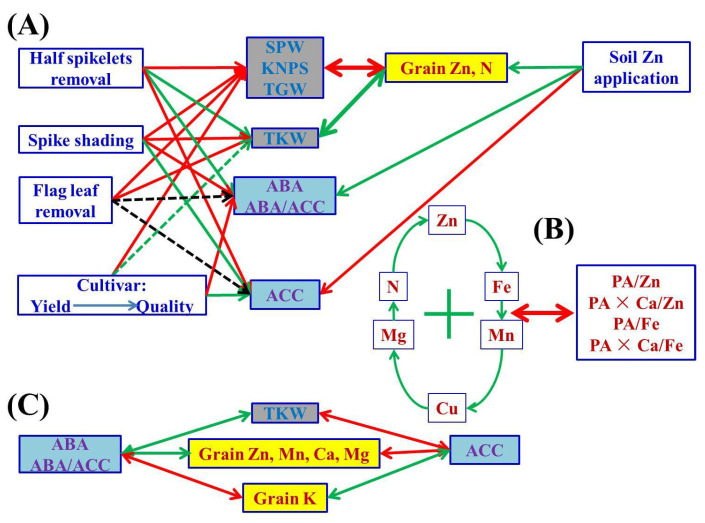
A schematic diagram summarizing possible cascades of grain phytohormones (concentrations of ABA and ACC, and ratios of ABA/ACC), grain yield traits and nutritional parameters (concentrations and ratios) of wheat crop affected by different soil Zn applications, cultivars and source–sink treatments. The one-way arrow in red indicates a decrease or negative influence, the one-way arrow in green indicates an increase or positive influence, and the two-way arrows in red and green colors indicate negative and positive correlation, respectively. “+” indicates mutual positive correlations among parameters. The dotted green line indicates weaker/less probability than the solid line; the black dotted line indicates an uncertain relationship. The abbreviations are as follows: single panicle weight (SPW), kernel number per spike (KNPS), thousand kernel weight (TKW), total grain weight (TGW), concentrations of abscisic acid (ABA), the ethylene precursor 1-aminocylopropane-1-carboxylic acid (ACC), Zn, Fe, Mn, Cu, N, P, K, Ca, Mg and phytate-P, ratios of C/N and ABA/ACC, and molar ratios of phytic acid (PA)/Zn, PA × Ca/Zn, PA/Fe and PA × Ca/Fe in wheat grains.

Our research showed that the application of Zn fertilizer (30 kg ZnSO_4_·7H_2_O ha^-1^) significantly increased grain yields/accumulation of N and K, and grain concentrations of Zn, N and K, but reduced Cu concentrations in wheat (Table 2 and Table 3, Tables S2 and S3). This is consistent with most previous studies on Zn [14,49,50], and consistent with Tao et al.’s results [48] on N. However, very few findings on the relationships between soil Zn application and grain accumulation of K or Cu have been reported in wheat plants; thus, the findings here require further verification. The simultaneous increase in grain concentrations of Zn and N could be explained by the interaction and co-localization of both within the grain, largely in the embryo and aleurone [51]. In general, rational Zn supply is beneficial for the improvement of grain Zn and protein/N nutrition for better human dietary quality (Figure 3).

### 3.2. Cultivars Showing Higher Grain Yields Had Lower Grain Protein and Micronutrient Nutritional Quality

The contradiction between grain nutritional quality and yield in crop breeding and production has been observed in many previous studies [14,26]. Breeders usually struggle with the 8–25% yield reduction because of low phytate wheat lines applied to enhance grain Zn bioavailability [52,53]. Although the “Green Revolution” since the 1960s and improved crop and soil management practices have increased the average grain yield of wheat more than two-fold [54,55,56,57], grain Zn concentrations have considerably decreased due to the so-called “dilution” effect [58,59,60,61]. Some results have demonstrated that grain Zn concentrations were negatively correlated with grain yields or cultivar release years among diverse wheat cultivars and regions [24,60]. The results in the current study also confirm this statement. The two wheat cultivars, Jimai 22 and Jimai 44, obviously differed in yield components (SPW, KNPS and TGW), grain concentrations of micronutrients, N, K and Ca, and bioavailability of Zn and Fe (Table 1, Table 2 and Table 3). The high-quality strong gluten wheat cultivar Jimai 44, with relatively lower SPW, KNPS and TGW than the most commonly used high-yielding Jimai 22, exhibited higher grain Zn, Mn, Cu and N concentrations and bioavailability of Zn and Fe, but lower grain concentrations of K and Ca. Grain Zn, Mn, Cu and N concentrations were negatively correlated with SPW, KNPS and/or TGW, whereas grain Ca concentrations and molar ratios of PA/Zn, PA × Ca/Zn and PA × Ca/Fe were positively correlated with SPW, KNPS and/or TGW (Figure 1). All of these results indicate that the higher the grain yields, the lower the grain micronutrient and N nutritional quality (Figure 3).

### 3.3. Effects of Physical Manipulation of Source/Sink on Grain Yield Traits and Nutrient Accumulation of Wheat

#### 3.3.1. Effects of Source–Sink Regulation on Grain Yields and Yield Components of Wheat

Many studies have reported the effects of source–sink treatments on wheat grain yields and/or yield components. Zhang et al. [17] showed that the defoliation (by removing all the leaf blades from tagged culms) and the spike shading significantly reduced the single grain weight of wheat at the mature stage, and the reduction caused by the defoliation was greater than the spike shading treatment. Half spikelet removal reduced the total sink capability and relatively increased the source strength, and slightly increased the single grain weight of each cultivar (0.3–7.0%) during the maturity period. Unlike Zhang et al. [17], the defoliation in the present study only removed the flag leaf, and this manipulation did not have the greatest impact on grain yield traits at the mature stage, with SPW, KNPS, TKW and TGW reduced by 22.7%, 11.4%, 14.2% and 24.1%, respectively (Table 1). The absence of a flag leaf would weaken photosynthesis and transpiration, and the production efficiency of carbohydrates to the grain would be reduced accordingly [62], but the remaining leaves could still accumulate dry matter to compensate [63]. Fu et al. [64] indicated that only removing the flag leaf after flowering had the least impact on grain yield composition compared to removing other leaves.

After the spike shading, the reductions in SPW (36.4%), KNPS (24.4%), TKW (16.9%) and TGW (37.0%) were greater than those from removing the flag leaf, which proved that the source of photosynthesis in the spike may be more important for grain filling and carbon assimilation accumulation than the effect of the flag leaf. Research showed that spike photosynthesis contributed 9.8–39.0% to wheat grain yield, with an average of 20.1% [65], the contribution of green leaves to grains was about 40%, and flag leaf accounted for about 19% [65,66]. Experiments have proved that after flowering, the panicle organs were in a favorable photosynthetic position, which was more conducive to intercepting light and CO_2_, especially in the process of leaves gradually losing photosynthetic capacity at the later stage of plant growth [67,68], indicating the importance of spike photosynthesis.

In our experiment, the SPW, KNPS and TGW were most severely reduced by 40.9%, 49.7% and 44.4%, respectively, whereas the TKW was significantly increased by 10.2%, after the removal of all spikelets from one side of the spike (i.e., half spikelet removal). Although appropriately reducing the sink capacity made the source–sink more coordinated, which was conducive to the increase in the single grain weight, this compensation effect still could not make up for the loss caused by excessive reduction in the KNPS [69].

#### 3.3.2. Effects of Source–Sink Regulation on Zn and Other Nutrient Concentrations in Wheat Grains

Previous studies reported that micronutrient concentrations in wheat grains were significantly reduced after defoliation [70], grain Zn, Fe, Mn and Cu concentrations increased to varying degrees after spikelet removal [18], and the concentration of trace elements also increased after spike shading [71]. In our study, we found that removing the flag leaf and spike shading decreased the grain Mn concentration, but the concentrations of Zn and Cu in wheat grains increased after spike shading (Table 2), suggesting that different elements were affected by the source to different degrees. Reducing the source of photosynthesis in the flag leaf and in non-leaf organs would reduce the supply of Mn and reduce the efficiency of transport to the grain. However, Zn, Cu and even Fe concentrations in the wheat grains in our experiment were less restricted by the source of photosynthesis. This may be due to the reduction in wheat photosynthesis production efficiency and changes in the accumulation of the chemical components of photosynthetic products, resulting in changes in the ratio of mineral metal elements [71], and ultimately increasing the concentration of most mineral metal elements in wheat grains. Consistent with previous studies [17,72], the grain Zn, Fe, Mn and Cu concentrations all increased after half spikelet removal. This may be due to the relative increase in the translocation amounts of nutrient elements received by the remaining grains [73]. Although the spikelet removal reduced the total sink, it relatively increased the source–sink ratio and supply of elements [17], and the carbohydrate supply level of the roots was also improved accordingly [74], which was beneficial to mineral nutrient absorption.

He et al. [75] investigated eight wheat cultivars and showed that the N concentration in the remaining wheat grains increased due to partial (25% or 50%) spikelet removal. By comparison, removal of the flag leaf or the upper two leaves reduced the uptake of N and P, and the grain N concentration. Flag leaf removal reduced the source–sink ratio, and the transpiration surface area and root carbohydrate levels decreased accordingly [75]. Therefore, the grain N concentration of the two cultivars also decreased after the flag leaf removal in our study (Table 3). The source–sink ratio decreased after the spike shading, and the source of photosynthesis also weakened, but the grain N and P concentrations increased significantly. This may be due to the fact that the supply of N and P had a weaker restriction on the concentrations of N and P in grains than that of carbohydrates on the starch content of grain [76,77,78]. By comparison, the half spikelet removal relatively increased the source–sink ratio and improved the carbohydrate supply level of the root system [79], which was beneficial to the increase in N and P concentrations in wheat grains in the current experiment (Table 3). Grain C/N ratios decreased accordingly in both treatments of spike shading and half spikelet removal.

Previous studies mainly focused on the effects of altered source–sink ratios on micronutrients, N and P of wheat, but rarely on the grain K, Ca and Mg. In our current study, we observed that grain Mg concentrations were increased by the flag leaf removal and half spikelet removal but decreased by spike shading. Grain K concentration was increased by spike shading but decreased by half spikelet removal. Grain Ca concentrations all decreased after spike shading and half spikelet removal (Table 3). The different responses of these three elements and corresponding underlying mechanisms need further verification and investigation.

Although concentrations of some nutrient elements increased in wheat grains, grain yields/accumulation of all nutrients (Zn, Fe, Mn, Cu, N, P, K, Ca and Mg) investigated in this study decreased to different extents due to the negative effects of source–sink regulation on grain yields and yield components (Table S3). In general, concentrations or yields of different elements were affected by the source–sink manipulation to different degrees.

#### 3.3.3. A Better Understanding of the “Dilution Effect” Caused by Yield Increase

Concentrations and yields of most nutrient elements in wheat grains were positively or negatively correlated with grain yield traits, indicating the strong link between them (Figure 1 and Figure S1). There may be a “dilution effect” between grain yield and grain micronutrient concentration, but previous research results were controversial, contradictory or inconsistent in terms of grain size, number or total grain yield. Grain Zn concentrations have been found to be correlated with thousand kernel weight negatively [20], positively [26], or not at all [23]. Grain Zn concentrations have been found to be correlated with grain numbers per m^2^ [20] or per pot [25], or spike numbers [26] negatively, but not with kernel numbers per spike [26]. Feil and Fossati [80] showed that there was a negative correlation between grain trace element concentration and grain yield. Calderini and Ortiz-Monasterio [81] reported that there was a positive correlation between grain mineral concentration and grain weight. In the present study, grain size and number and total grain yield were greatly affected by different source–sink manipulations; SPW, KNPS and TGW consistently showed negative correlations with grain concentrations of Zn, N and Mg, whereas the impact of TKW was the exact opposite (Figure 1). The current results indicate that the dilution effect occurred due to the increase in grain number and total grain yield, and the “enrichment effect” we propose here for the first time occurred due to the increase in grain size or single kernel/grain weight (Figure 3). Therefore, the weight/size and nutrient accumulation per grain/kernel could increase synchronously, i.e., an enrichment effect, which can be used to distinguish from the term “dilution effect” to avoid controversy for better understanding of the relationship between nutrient accumulation and grain yield traits.

#### 3.3.4. Effects of Source–Sink Regulation on the Bioavailability of Zn and Fe in Wheat Grains

In addition to the concentration of Zn and Fe in grains, their bioavailability (estimated by molar ratios of PA/Zn, PA/Fe, PA × Ca/Zn and PA × Ca/Fe) is also crucial for increasing the human body’s daily Zn intake [26]. A molar ratio of PA/Zn and PA × Ca/Zn lower than the critical ratios of 15 and 200, respectively, indicate better Zn bioavailability [36,37], but the molar ratio of PA/Zn in most cereal products ranges from 25 to 34 [82]. The critical value of the PA/Fe molar ratio is 10 [38]. In the present study, we observed for the first time that the concentration and, in particular, the bioavailability of Zn or Fe in wheat grains could be simultaneously improved by half spikelet removal and spike shading, but not by flag leaf removal (Table 2 and Table 3). However, the resulting molar ratios were still much higher than their critical values, indicating the bioavailability of Zn and Fe was not improved sufficiently. This could be related to the simultaneous increase in phytate-P and Zn/Fe after half spikelet removal and spike shading, because PA was closely bound to Zn/Fe to form spherical crystals with a poorly soluble protein structure [83], which had strong binding force to Zn/Fe, thus reducing the dissolution rate of Zn/Fe. In our previous study, foliar Zn spraying significantly improved wheat grain Zn concentration, but the anti-nutritional compound phytate-P concentration was less affected, so molar ratios of PA/Zn below 15.0 and PA × Ca/Zn below 200 occurred, suggesting higher Zn bioavailability [26]. Although the concentrations of Zn and Fe were substantially increased and Ca was also decreased to some extent, the simultaneously increased PA limited the increase in bioavailability of Zn and Fe in the current study (Table 2 and Table 3). Therefore, to achieve the target level of biofortification, it is critical to not only significantly increase micronutrient concentrations in wheat grains, but also to aim to regulate the concentration of PA appropriately (e.g., breeding low phytate wheat cultivars), to finally improve the bioavailability of Zn and Fe in food [84,85].

### 3.4. Phytohormones (ABA and ACC) Involved in Nutrient and Biomass Accumulation in Wheat Grains

Although the relationship between phytohormones and homeostasis of various nutrient elements in crop plants has been rarely studied, there are still several indications that phytohormones may participate in the source–sink interaction of elemental nutrition (e.g., Zn) in wheat plants. For example, when wheat leaves were exposed to nano-ZnO stress, the photosynthetic carbon assimilation and antioxidant capacity were improved by melatonin [86]. The manipulation of cytokinin dehydrogenase (CKX, the enzyme that inactivates cytokinin) clearly impacts root growth and orientation, yield and grain Zn nutrition in cereals [87]. Higher ABA levels and ratios of ABA/GAs and ABA/ethylene were required for the efficient grain-filling of wheat in the report of Yang and Zhang [13]. In the present study, it appears that the elevated endogenous ABA levels and ABA/ACC ratios promoted the TKW and the grain-filling of Zn, Mn, Ca and Mg, but inhibited K in wheat grains (Figure 1 and Figure 3). There were positive correlations between grain ABA concentrations or ABA/ACC ratios and TKW, and grain concentrations of Zn, Mn, Ca or Mg. However, the effects of ACC were diametrically opposite. To our knowledge, this is the first report on such phenomena, which need further in-depth verification and investigation, particularly to provide direct experimental evidence between ABA or ACC and homeostasis genes/binding proteins or transcripts associated with efficient transport and accumulation of nutrient elements in wheat plants, and to ascertain whether the exogenous ABA can be applied to improve wheat grain nutritional quality, which would shed new light on biofortification.

## 4. Materials and Methods

### 4.1. Study Site

The field experiment was conducted during the 2018–2019 growing season at Jinan Licheng Experimental Station (36°42′39″ N, 117°4′39″ E), Crop Research Institute, Shandong Academy of Agricultural Sciences, China. The area has a typical continental and warm climate, with an annual mean temperature of 14.7 °C and a long-term mean annual rainfall of 671.1 mm. The soil at the site was classified as clay loam, with a pH of 7.8 (1:2.5 *w*/*v* in water). The top 20 cm of the soil contained 21 g kg^−1^ organic matter, 79 mg kg^−1^ water-hydrolysable N, 27 mg·kg^−1^ Olsen-P, 178 mg·kg^−1^ exchangeable K and 1.6 mg·kg^−1^ DTPA-extractable Zn.

### 4.2. Experimental Design

The experiment was a split-split-plot design with three factors consisting of four source–sink treatments (split-split plot), two wheat cultivars (subplot) and two soil Zn application levels (main plot) in three replicates. Two application levels of Zn fertilizer to soil were set: (1) no Zn application (zero); (2) 30 kg·ha^−1^ ZnSO_4_·7H_2_O. The two winter wheat (*Triticum aestivum* L.) cultivars were Jimai 22 and Jimai 44. Jimai 22 is a high-yielding wheat cultivar and is sown over the largest area in contemporary China. Jimai 44 is a high-quality strong gluten wheat cultivar, suitable for making bread. The four source–sink treatments included: (1) no treatment as a control (CK); (2) flag leaf removal, removing the flag leaf blade from a tagged culm [88]; (3) half spikelet removal—all spikelets were removed from one side of the marked spike [89]; and (4) spike shading, wrapping the marked spike with aluminum foil paper (there were several micro-holes less than 1 mm^2^ in the aluminum foil paper to facilitate the exchange of internal and external gas) [17]. The area of the main plot was 25 × 100 m = 2500 m^2^, that of the subplot was 25 × 22 m = 550 m^2^, and that of the split-split plot was 2 × 2.5 m = 5.0 m^2^.

The wheat sowing date was 22 October 2018 and harvest date was 7 June 2019. Thirty spikes of wheat plants that flowered on the same day were tagged for later treatments in each plot. Source–sink manipulations were conducted 5 days after flowering. At maturity, all wheat spikes treated/tagged in each plot were removed. Grains from two sampled spikes were then immediately ground into fine powder in liquid nitrogen for measurements of abscisic acid (ABA) and the ethylene precursor 1-aminocylopropane-1-carboxylic acid (ACC). The other 28 wheat spikes left were used for detailed investigation on the grain yield (TGW: total grain weight) and yield component (SPW: single panicle weight, KNPS: kernel number per spike and TKW: thousand kernel weight), and for nutrient analysis. All grains were manually separated from the husks.

A quantity of 750 kg·ha^−1^ of the compound fertilizer (NPK 15-15-15) and Zn fertilizer (0 or 30 kg·ha^−1^ ZnSO_4_·7H_2_O) were evenly distributed and incorporated into the upper 20 cm of the soil prior to wheat planting. The other 112.5 kg of N ha^−1^ (supplied as urea) was top-dressed with irrigation at the jointing stage. All plots were adequately irrigated at stages of pre-wintering, stem elongation and flowering, and weeded manually. There were no fungicides applied during the growth period. At the booting stage, omethoate (2-dimethoxyphosphinoylthio-N-methylacetamide) (Dazhou Xinglong Chemical Co., Ltd., Dazhou, China) was sprayed to control aphids.

### 4.3. Quantification of ABA and ACC

The extraction and quantification of phytohormones for liquid chromatography-tandem mass spectrometry (LC-MS/MS) analysis were described previously [90,91,92]. The determination of ABA and ACC in this study was carried out at Shanghai Applied Protein Technology Company (Shanghai, China).

### 4.4. Nutrient Analysis

After the wheat was threshed, grain samples were quickly rinsed with deionized water, dried in an oven at 60–65 °C for 72 h, and then ground with a stainless-steel grinder (RT-02B, Chinese Taipei). Ground samples were digested with HNO_3_-H_2_O_2_ in a closed microwave digester (CEM, Matthews, NC, USA). The concentrations of nutrients (Zn, Fe, Mn, Cu, Ca, Mg, P and K) in the digests were determined by inductively coupled plasma atomic emission spectroscopy (ICP-AES, OPTIMA 3300 DV, PerkinElmer, Waltham, MA, USA). Two blanks and a standard grain sample Henan wheat GBW 10046 (GSB-24) were included in each batch to ensure analytical quality. The N concentration of grain was determined by the H_2_SO_4_-H_2_O_2_ digestion-Kjeldahl method. The ratio of C/N was determined using Multi N/C 3100 (Analytik Jena AG, Gina, Germany). Phytate-P concentration was analyzed according to the method of Haug and Lantzsch [93]. Phytate-P was converted to PA by dividing by 0.282. The nutrient concentration of PA, Zn, Fe or Ca (in the unit of g kg^−1^ or mg kg^−1^) was converted to the molar number per kilogram grain by dividing by its molecular weight, to calculate the molar ratios of PA/Zn, PA × Ca/Zn, PA/Fe and PA × Ca/Fe, which were used to predict the bioavailability of Zn and Fe in wheat grains.

### 4.5. Statistical Analysis

Data were subjected to ANOVA using SAS software (SAS 8.0, SAS Institute, Cary, NC, USA) and means were compared by Fisher’s protected least significant difference (LSD) at *p* ≤ 0.05. The relationships among all the investigated parameters of wheat plants were determined by Pearson’s correlation analysis performed by OriginPro 2021. Principal component analysis (PCA) was also performed to compare the effect of source–sink manipulation on the various parameters of wheat investigated.

## 5. Conclusions

A schematic diagram summarizing possible cascades of grain phytohormones (concentrations of ABA and ACC, and ratios of ABA/ACC), grain yield traits and nutritional parameters (concentrations and ratios) of wheat crop affected by different soil Zn applications, cultivars and source–sink treatments is shown in Figure 3. After soil application of Zn, there were no significant changes in grain yield components of wheat, but the grain Zn and N concentrations increased simultaneously. In general, a rational Zn supply is beneficial for the improvement of grain Zn and protein/N nutrition for better human dietary quality. Cultivars showing higher grain yields had lower grain protein and micronutrient nutritional quality. The SPW, KNPS, TGW and concentrations of grain K and Ca of the most commonly used high-yielding cultivar, Jimai 22, were significantly higher than those of the high-quality strong gluten wheat cultivar, Jimai 44, whereas the grain concentrations of Zn, Mn and Cu, and bioavailability of Zn and Fe of Jimai 22 were significantly lower than those of Jimai 44. Grain size, number, total grain yield and nutrient accumulation were greatly affected by different source–sink manipulations. SPW, KNPS, TKW (with the exception of TKW in half spikelet removal), TGW and nutrient accumulation in wheat grains were most severely reduced by half spikelet removal and less by spike shading, with flag leaf removal generally having the least impact. Grain concentrations of Zn, N and Mg consistently showed negative correlations with SPW, KNPS and TGW, but positively with TKW. There were general positive correlations among grain concentrations of Zn, Fe, Mn, Cu, N and Mg, and most correlations between these nutrient concentrations and molar ratios of PA/Zn, PA/Fe, PA × Ca/Zn and PA × Ca/Fe were negative. Although the concentrations of Zn and Fe were substantially increased and Ca was also decreased to some extent in treatments of half spikelet removal and spike shading, the simultaneously increased PA limited the increase in bioavailability of Zn and Fe. There were also positive or negative correlations among the other nutrient elements in wheat grains. In general, different nutrient elements interact with each other and are affected to different degrees by source–sink manipulations. Phytohormones (ABA and ACC) are involved in nutrient and biomass accumulation in wheat grains. It appears that the elevated endogenous ABA levels and ABA/ACC ratios promoted the TKW and the grain-filling of Zn, Mn, Ca and Mg, but inhibited K in wheat grains. These results provide better understanding of source–sink relationships of Zn and other nutrient elements for wheat grain biofortification to alleviate human malnutrition. The underlying molecular and physiological regulatory mechanisms under different source–sink manipulations need to be further studied.

## Figures and Tables

**Table 1:** Effects of soil Zn application and source–sink manipulations on grain yields and yield components of different wheat cultivars.

Treatments	Single Panicle Weight (g)	Kernel Number Per Spike	Thousand Kernel Weight (g)	Total Grain Weight (g)
**Zn Application Rate (kg** **·ha^–1^)**				
ZnSO_4˙_7H_2_O (0)	1.6	30.4	42.5	38.3
ZnSO_4˙_7H_2_O (30)	1.6	30.3	42.9	38.5
LSD_0.05_	0.1	1.2	1.6	2.5
**Cultivar (C)**				
Jimai 22	1.7a	32.4a	42.4	40.6a
Jimai 44	1.5b	28.3b	43.0	36.2b
LSD_0.05_	0.1	1.2	1.6	2.5
**Source–Sink Treatment (SS)**				
Control	2.2a	38.6a	45.1b	52.2a
Flag leaf removal	1.7b	34.2b	38.7c	39.6b
Half spikelets removal	1.3d	19.4d	49.7a	29.0d
Spike shading	1.4c	29.2c	37.5c	32.9c
LSD_0.05_	0.1	1.7	2.3	3.5
**ANOVA**				
Zn	0.6016	0.9573	0.6063	0.8436
C	<0.0001	<0.0001	0.4486	0.0009
SS	<0.0001	<0.0001	<0.0001	<0.0001
Zn × C	0.8687	0.9866	0.3948	0.6383
Zn × SS	0.1414	0.0213	0.4815	0.1067
C × SS	0.0074	0.0649	0.1021	0.0942
Zn × C × SS	0.3890	0.3733	0.6299	0.4968

Total grain weight is the sum grain weight of 28 wheat spikes sampled in each plot. Values followed by different lowercase letters in the same column are significantly different among treatments at *p* ≤ 0.05. Values under ANOVA are probabilities (*p* values) of the source of variation.

**Table 2:** Effects of soil Zn application and source–sink manipulations on concentrations of Zn, Fe, Mn and Cu in grains of different wheat cultivars.

Treatments	Zn	Fe	Mn	Cu
	(mg·kg^−1^)			
**Zn application rate (kg·ha^−1^)**				
ZnSO_4˙_7H_2_O (0)	41.1b	49.8	46.4	5.6a
ZnSO_4˙_7H_2_O (30)	43.2a	42.5	46.2	5.3b
LSD_0.05_	2.0	9.2	2.2	0.2
**Cultivar (C)**				
Jimai 22	40.6b	44.8	45.1b	5.1b
Jimai 44	43.7a	47.5	47.5a	5.8a
LSD_0.05_	2.0	9.2	2.2	0.2
**Source–Sink Treatment (SS)**				
Control	35.8c	42.1	45.4b	4.9b
Flag leaf removal	35.7c	44.1	41.2c	5.0b
Half spikelet removal	53.2a	52.8	59.6a	5.9a
Spike shading	43.6b	45.6	39.0c	6.0a
LSD_0.05_	2.9	13.0	3.2	0.3
**ANOVA**				
Zn	0.0420	0.1125	0.8720	0.0007
C	0.0040	0.5612	0.0332	<0.0001
SS	<0.0001	0.3744	<0.0001	<0.0001
Zn × C	0.0417	0.3430	0.4459	0.0148
Zn × SS	0.7241	0.9119	0.3553	0.2445
C × SS	<0.0001	0.4729	0.2124	0.0469
Zn × C × SS	0.0449	0.4959	0.3544	0.1657

Values followed by different lowercase letters in the same column are significantly different among treatments at *p* ≤ 0.05. Values under ANOVA are probabilities (*p* values) of the source of variation.

**Table 3:** Effects of soil Zn application and source–sink manipulations on concentrations of N, P, K, Ca, Mg and phytate-P, C/N ratios, and molar ratios of PA/Zn, PA × Ca/Zn, PA/Fe and PA × Ca/Fe in grains of different wheat cultivars.

Treatments	N	P	K	Ca	Mg	C/N	Phytate-P	PA/Zn	PA × Ca/Zn	PA/Fe	PA × Ca/Fe
	(g·kg^−1^)	(g·kg^−1^)	(g·kg^−1^)	(mg·kg^−1^)	(mg·kg^−1^)		(g·kg^−1^)				
**Zn Application Rate (kg** **·ha^−1^)**											
ZnSO_4˙_7H_2_O (0)	17.1b	3.4	3.8b	458.9	1533.9	26.3	3.1	30.3	348.0	22.7	261.9
ZnSO_4˙_7H_2_O (30)	19.2a	3.3	4.3a	461.3	1566.0	24.7	3.2	29.1	336.1	25.6	294.8
LSD_0.05_	0.5	0.4	0.2	10.9	54.2	1.8	0.2	2.1	25.8	3.4	41.6
**Cultivar (C)**											
Jimai 22	17.8b	3.2	4.3a	474.7a	1543.3	26.0	3.2	31.3a	371.2a	25.9a	307.1a
Jimai 44	18.5a	3.4	3.8b	445.5b	1556.6	25.0	3.1	28.0b	312.9b	22.4b	249.6b
LSD_0.05_	0.5	0.4	0.2	10.9	54.2	1.8	0.2	2.1	25.8	3.4	41.6
**Source–Sink Treatment (SS)**											
Control	17.0c	2.6b	3.6b	495.2a	1439.2c	27.4a	2.8c	31.0a	383.4a	23.4	289.8ab
Flag leaf removal	16.2d	2.9b	3.9b	491.7ab	1524.5b	27.6a	2.9c	32.1a	393.9a	25.6	315.8a
Half spikelets removal	20.1a	4.0a	3.2c	477.5b	1820.7a	24.0b	3.6a	26.1b	312.8b	22.8	274.3ab
Spike shading	19.2b	3.8a	5.5a	376.0c	1415.3c	23.0b	3.3b	29.7a	278.7b	24.9	233.6b
LSD_0.05_	0.7	0.5	0.3	15.4	76.7	2.5	0.2	3.3	39.1	4.8	58.9
**ANOVA**											
Zn	<0.0001	0.6780	0.0007	0.6502	0.2367	0.0751	0.3775	0.2692	0.3507	0.0917	0.1176
C	0.0157	0.1611	0.0003	<0.0001	0.6197	0.2441	0.3197	0.0032	<0.0001	0.0421	0.0085
T	<0.0001	<0.0001	<0.0001	<0.0001	<0.0001	0.0006	<0.0001	0.0023	<0.0001	0.6304	0.0540
Zn × C	0.5808	0.0532	0.1328	0.0883	0.4575	0.2489	0.8534	0.1911	0.1263	0.5354	0.3964
Zn × SS	0.4284	0.1946	0.2364	0.0004	0.2855	0.3302	0.4835	0.5974	0.8836	0.8139	0.9642
C × SS	0.1999	0.0593	0.0084	0.0064	0.1488	0.0592	0.0837	0.2684	0.5623	0.9104	0.9586
Zn × C × SS	0.3281	0.4752	0.3643	0.0695	0.5977	0.4837	0.1155	0.0274	0.0538	0.7255	0.6029

Values followed by different lowercase letters in the same column are significantly different among treatments at *p* ≤ 0.05. Values under ANOVA are probabilities (*p* values) of the source of variation.

**Table 4:** Effects of soil Zn application and source–sink manipulations on concentrations of ABA and ACC and ratios of ABA/ACC in grains of different wheat cultivars.

Treatments	ABA (ng·g^−1^)	ACC (ng·g^−1^)	ABA/ACC
**Zn application rate (kg·ha^−1^)**			
ZnSO_4˙_7H_2_O (0)	25.2b	41.0a	0.7b
ZnSO_4˙_7H_2_O (30)	31.0a	35.3b	1.0a
LSD_0.05_	6.3	5.2	0.2
**Cultivar (C)**			
Jimai 22	30.9	32.6b	1.1a
Jimai 44	25.3	43.7a	0.7b
LSD_0.05_	6.3	5.2	0.2
**Source–Sink treatment (SS)**			
Control	28.4b	34.4b	0.9b
Flag leaf removal	25.8b	34.5b	0.8b
Half spikelet removal	42.6a	30.9b	1.5a
Spike shading	15.4c	52.7a	0.3c
LSD_0.05_	9.0	7.4	0.3
**ANOVA**			
Zn	0.0700	0.0349	0.0152
C	0.0811	0.0002	0.0029
SS	<0.0001	<0.0001	<0.0001
Zn × C	0.2616	0.0125	0.2208
Zn × SS	0.2612	0.6570	0.0634
C × SS	0.1900	0.7373	0.1347
Zn × C × SS	0.5728	0.3375	0.8401

Values followed by different lowercase letters in the same column are significantly different among treatments at *p* ≤ 0.05. Values under ANOVA are probabilities (*p* values) of the source of variation.

## Data Availability

Not applicable.

## Supplementary Materials

The following are available online at https://www.mdpi.com/article/10.3390/plants10051032/s1, Table S1: Effects of soil Zn application and source–sink manipulations on single panicle weights and kernel numbers per spike of different wheat cultivars, Table S2: Effects of soil Zn application and source–sink manipulations on concentrations of Zn, Cu, Ca and K in grains of different wheat cultivars, Table S3: Effects of soil Zn application and source–sink manipulations on yields of Zn, Fe, Mn, Cu, N, P, K, Ca, Mg and phytate-P in grains of 28 wheat spikes sampled in each plot, Table S4: Effects of soil Zn application and source–sink manipulations on yields of ABA and ACC in grains of 28 wheat spikes sampled in each plot, Figure S1: Corrplot representing correlation among measured grain phytohormones (yields of ABA and ACC, and ratios of ABA/ACC), grain yield traits and nutritional parameters (yields and ratios) of wheat crop across different soil Zn applications, cultivars and source–sink treatments (*n* = 48).
