# Supplementary material for: Source–Sink Manipulation Affects Accumulation of Zinc and Other Nutrient Elements in Wheat Grains
Source: Plants (Basel). 2021 May 20;10(5):1032. doi: 10.3390/plants10051032 (PMC8161399; doi:10.3390/plants10051032)
Supplement: Supplementary file 1 [file plants-10-01032-s001.zip › plants-1176131-supplementary.pdf]

SUPPLEMENTARY MATERIALS

Table S1. Effects of soil Zn application and source-sink manipulations on single panicle weights and kernel numbers per spike of different wheat cultivars.

Zn application rate ($ZnSO_4 \cdot 7H_2O$ kg·ha^{-1})	Treatments	Single panicle weight (g)		Kernel number per spike	
		**Jimai 22**	**Jimai 44**	**Jimai 22**	**Jimai 44**
0	Control	2.5A	2.0A	42.2A	34.5AB
	Flag leaf removal	1.7B	1.7B	36.0Ba	31.3BCb
	Half spikelets removal	1.3D	1.2C	19.9D	17.2E
	Spike shading	1.6BC	1.3C	31.7C	30.2C
30	Control	2.4A	2.1A	41.9A	35.7A
	Flag leaf removal	1.7B	1.6B	35.9B	33.6ABC
	Half spikelets removal	1.4Ca	1.3Cb	21.8D	18.6E
	Spike shading	1.4C	1.2C	29.9C	25.2D

Values followed by different capital letters in the same column are significantly different among treatments at $p \leq 0.05$. Values followed by different lowercase letters in the same row are significantly different among treatments at $p \leq 0.05$.

Table S2. Effects of soil Zn application and source-sink manipulations on concentrations of Zn, Cu, Ca and K in grains of different wheat cultivars.

Zn application rate ($ZnSO_4·7H_2O$ kg·ha^{-1})	Treatments	Zn (mg·kg^{-1})		Cu (mg·kg^{-1})		Ca (mg·kg^{-1})		K (g·kg^{-1})	
		Jimai 22	Jimai 44	Jimai 22	Jimai 44	Jimai 22	Jimai 44	Jimai 22	Jimai 44
0	Control	29.2D	42.2BC	4.4B	5.4CD	497.4ABC	484.5AB	3.8BC	3.2CD
	Flag leaf removal	35.0C	34.3D	4.6Bb	5.7CDa	473.5C	481.1AB	4.0B	3.4C
	Half spikelets removal	55.6Aa	47.4ABb	5.9Ab	6.2Ba	487.4BC	453.9BC	3.1C	2.9D
	Spike shading	42.4B	42.4BC	5.8A	6.9A	416.8D	376.3D	5.7A	4.6B
30	Control	32.4CD	39.5CD	4.3B	5.3DE	518.6AB	480.3AB	3.9BC	3.4C
	Flag leaf removal	33.3Cb	40.8BCDa	4.7B	4.9E	513.4AB	498.8A	3.8BC	4.2B
	Half spikelets removal	56.4A	53.4A	5.6Ab	5.8BCa	528.8Aa	439.8Cb	3.4BC	3.4C
	Spike shading	40.2B	49.5A	5.6A	5.8BC	361.6E	349.1D	6.5A	5.4A

Values followed by different capital letters in the same column are significantly different among treatments at $p \leq 0.05$. Values followed by different lowercase letters in the same row are significantly different among treatments at $p \leq 0.05$.

Table S3. Effects of soil Zn application and source-sink manipulations on yields of Zn, Fe, Mn, Cu, N, P, K, Ca, Mg and phytate-P in grains of 28 wheat spikes sampled in each plot.

Treatments	Zn (mg)	Fe (mg)	Mn (mg)	Cu (mg)	N (mg)	P (mg)	K (mg)	Ca (mg)	Mg (mg)	Phytate-P (mg)
**Zn application rate (kg·ha^{-1})**										
$ZnSO_4{\cdot}7H_2O$ (0)	1.5	1.9	1.7	0.21	644.4b	123.4	147.1b	17.7	57.6	116.4
$ZnSO_4{\cdot}7H_2O$ (30)	1.6	1.6	1.8	0.20	724.3a	120.0	161.0a	18.2	59.8	119.3
$LSD_{0.05}$	0.1	0.4	0.1	0.02	44.7	13.9	11.5	1.3	4.4	9.0
**Cultivar (C)**										
Jimai 22	1.6	1.8	1.8	0.20	708.7a	125.8	170.6a	19.5a	61.8a	125.5a
Jimai 44	1.6	1.7	1.7	0.21	660.1b	117.6	137.5b	16.4b	55.6b	110.2b
$LSD_{0.05}$	0.1	0.4	0.1	0.02	44.7	13.9	11.5	1.3	4.4	9.0
**Source-sink treatment (SS)**										
Control	1.8a	2.2a	2.4a	0.25a	882.5a	135.8a	187.5a	25.9a	74.9a	143.5a
Flag leaf removal	1.4b	1.8ab	1.6b	0.20b	642.4b	111.9b	153.1b	19.5b	60.4b	115.6b
Half spikelets removal	1.6b	1.5b	1.7b	0.17c	584.4b	115.0b	93.4c	13.9c	52.9c	104.1b
Spike shading	1.4b	1.5b	1.3c	0.20b	628.1b	124.3ab	182.1a	12.4c	46.6d	108.2b
$LSD_{0.05}$	0.2	0.6	0.2	0.02	63.2	19.6	18.3	1.9	6.2	12.8
**ANOVA**										
Zn	0.2347	0.1907	0.6355	0.1680	0.0010	0.6175	0.0198	0.4889	0.3213	0.5164
C	0.7439	0.6408	0.0753	0.6930	0.0342	0.2355	<0.0001	<0.0001	0.0067	0.0017
SS	0.0003	0.1216	<0.0001	<0.0001	<0.0001	0.0782	<0.0001	<0.0001	<0.0001	<0.0001
Zn × C	0.3535	0.3391	0.9158	0.4711	0.6835	0.0534	0.2493	0.9567	0.4253	0.6445
Zn × SS	0.1772	0.8502	0.0873	0.0645	0.0628	0.6066	0.4695	0.0209	0.0696	0.2355
C × SS	0.0056	0.6374	0.0257	0.1608	0.2828	0.0641	0.0144	0.0330	0.1193	0.1637
Zn × C × SS	0.5445	0.4791	0.2593	0.2293	0.2742	0.6632	0.1987	0.8720	0.5871	0.0594

Values followed by different lowercase letters in the same column are significantly different among treatments at $p \leq 0.05$. Values under ANOVA are probabilities (p values) of the source of variation.

Table S4. Effects of soil Zn application and source-sink manipulations on yields of ABA and ACC in grains of 28 wheat spikes sampled in each plot.

Treatments	ABA (ng)	ACC (ng)
**Zn application rate (kg·ha^{-1})**		
$ZnSO_4 \cdot 7H_2O$ (0)	961.6	1529.6a
$ZnSO_4 \cdot 7H_2O$ (30)	1182.3	1322.2b
$LSD_{0.05}$	254.2	185.7
**Cultivar (C)**		
Jimai 22	1256.8a	1290.6b
Jimai 44	887.2b	1561.2a
$LSD_{0.05}$	254.2	185.7
**Source-sink treatment (T)**		
Control	1485.8a	1741.7a
Flag leaf removal	1031.2b	1355.3b
Half spikelets removal	1260.1ab	875.5c
Spike shading	510.8c	1731.0a
$LSD_{0.05}$	359.5	262.7
**ANOVA**		
Zn	0.0863	0.0299
C	0.0058	0.0057
SS	<0.0001	<0.0001
Zn × C	0.2961	0.0139
Zn × SS	0.2433	0.5183
C × SS	0.0233	0.3574
Zn × C × SS	0.6095	0.1047

Values followed by different lowercase letters in the same column are significantly different among treatments at $p \leq 0.05$. Values under ANOVA are probabilities (p values) of the source of variation.

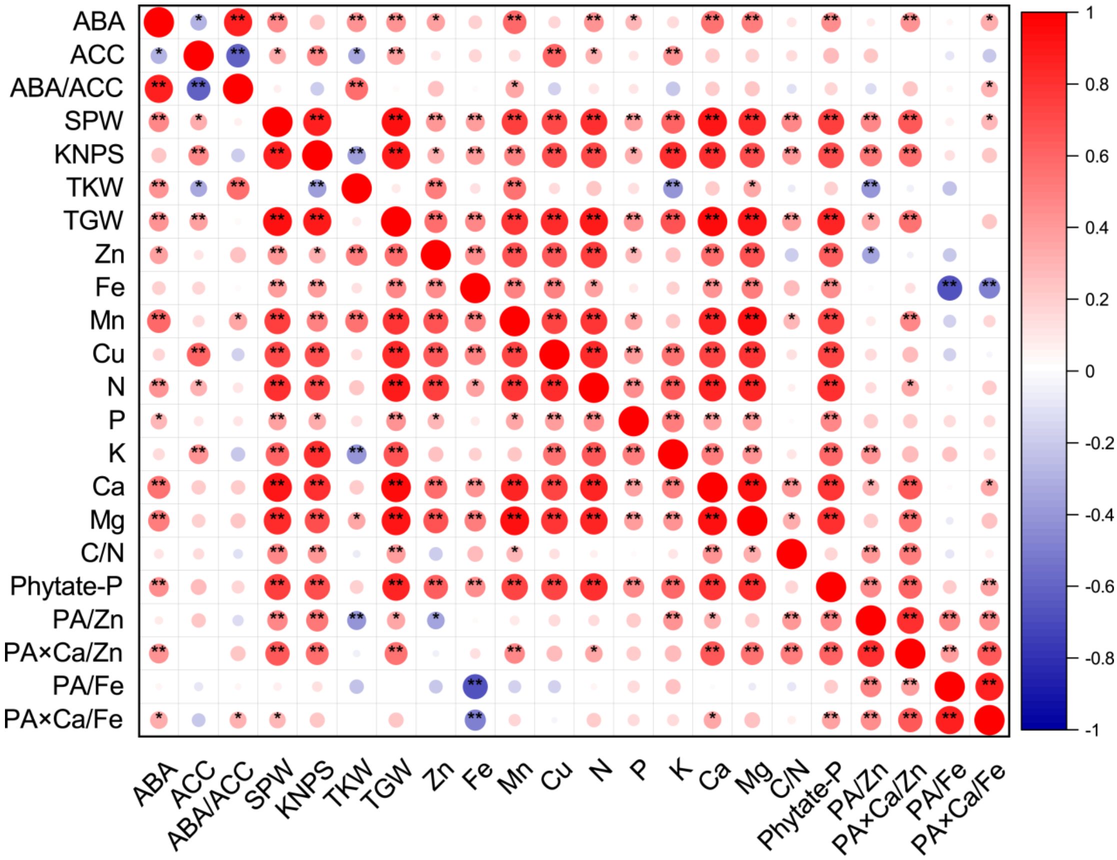

Figure S1. Corrplot representing correlation among measured grain phytohormones (yields of ABA and ACC, and ratios of ABA/ACC), grain yield traits and nutritional parameters (yields and ratios) of wheat crop across different soil Zn applications, cultivars and source-sink treatments (n = 48). Positive correlations are displayed in red and negative correlations in blue color. The color legend on the right-hand side of corrplot, shows correlation coefficients and the corresponding colors. The intensity of the color and circle size is proportional to the correlation coefficients. "*" and "**" indicate significant correlations at $p \leq 0.05$ and $p \leq 0.01$, respectively. The abbreviations are as follows: single panicle weight (SPW), kernel number per spike (KNPS), thousand kernel weight (TKW), total grain weight (TGW), yields/accumulation of abscisic acid (ABA), the ethylene precursor 1-aminocylopropane-1-carboxylic acid (ACC), Zn, Fe, Mn, Cu, N, P, K, Ca, Mg and phytate-P, ratios of C/N and ABA/ACC, and molar ratios of phytic acid (PA)/Zn, PA × Ca/Zn, PA/Fe and PA × Ca/Fe in wheat grains.
